# Anaesthesia and airway management in mucopolysaccharidosis

**DOI:** 10.1007/s10545-012-9563-1

**Published:** 2012-11-30

**Authors:** Robert Walker, Kumar G. Belani, Elizabeth A. Braunlin, Iain A. Bruce, Henrik Hack, Paul R. Harmatz, Simon Jones, Richard Rowe, Guirish A. Solanki, Barbara Valdemarsson

**Affiliations:** 1 Royal Manchester Children’s Hospital, Oxford Road, Manchester, M13 9WL UK; 2 University of Minnesota, Minneapolis, MN USA; 3 Children’s Hospital and Research Center Oakland, Oakland, CA USA; 4 St Mary’s Hospital, Manchester, UK; 5 Birmingham Children’s Hospital, Birmingham, UK; 6 Queen Sylvia’s Children Hospital, Gothenburg, Sweden

## Abstract

This paper provides a detailed overview and discussion of anaesthesia in patients with mucopolysaccharidosis (MPS), the evaluation of risk factors in these patients and their anaesthetic management, including emergency airway issues. MPS represents a group of rare lysosomal storage disorders associated with an array of clinical manifestations. The high prevalence of airway obstruction and restrictive pulmonary disease in combination with cardiovascular manifestations poses a high anaesthetic risk to these patients. Typical anaesthetic problems include airway obstruction after induction or extubation, intubation difficulties or failure [can’t intubate, can’t ventilate (CICV)], possible emergency tracheostomy and cardiovascular and cervical spine issues. Because of the high anaesthetic risk, the benefits of a procedure in patients with MPS should always be balanced against the associated risks. Therefore, careful evaluation of anaesthetic risk factors should be made before the procedure, involving evaluation of airways and cardiorespiratory and cervical spine problems. In addition, information on the specific type of MPS, prior history of anaesthesia, presence of cervical instability and range of motion of the temporomandibular joint are important and may be pivotal to prevent complications during anaesthesia. Knowledge of these risk factors allows the anaesthetist to anticipate potential problems that may arise during or after the procedure. Anaesthesia in MPS patients should be preferably done by an experienced (paediatric) anaesthetist, supported by a multidisciplinary team (ear, nose, throat surgeon and intensive care team), with access to all necessary equipment and support.

**Electronic supplementary material:**

The online version of this article (doi:10.1007/s10545-012-9563-1) contains supplementary material, which is available to authorized users.

## Introduction to mucopolysaccharidosis (MPS)

The mucopolysaccharidoses (MPS) are inherited lysosomal storage diseases associated with accumulation of glycosaminoglycans (GAGs) in tissues and organs. They are associated with progressively worsening organ dysfunction that eventually leads to a decreased lifespan. Typical clinical manifestations include coarse facial features, ear–nose–throat (ENT) problems, skeletal dysplasia, growth impairment, cervical instability and spinal cord compression, organomegaly, impaired vision and hearing, joint contractures, hernias and cardiorespiratory disease (Muenzer 2011). Some types are associated with cognitive impairment [MPS IH (MIM ID #607014), II (MIM ID #309900), III (MIM ID #252900/20/30/40) and VII (MIM ID #253220)] or increased joint mobility [MPS IVA (MIM ID #253000)]. Clinical progression rates within the different forms of MPS vary considerably.

Most MPS patients require anaesthesia for multiple surgical interventions to help manage the disease. Data from the MPS I registry (*N* = 544) showed at least one surgical procedure in 75 % of patients, with a median of three to four surgeries per patient (Arn et al. 2009). Data from the Hunter Outcome Survey (*N* = 527) showed surgical interventions in 83.7 % of MPS II patients (Mendelsohn et al. 2010). Anaesthesia for magnetic resonance imaging (MRI) is frequently needed in very young children and older MPS patients with developmental delay. MPS patients can have an increased anaesthetic risk due to a difficult airway, cervical spine disease and an increased prevalence of cardiovascular manifestations (Kamin 2008; Wold et al. 2010).

## Anaesthetic complications and surgical mortality in MPS patients

Surgery in MPS patients is associated with a high mortality rate. A study comprising 932 patients enrolled in the MPS I registry that underwent a total of 4,762 procedures showed 30-day risk of death/procedure and death/patient rates of 0.7 % and 4.2 % (Arn et al. 2012). Most serious anaesthetic complications occurring during surgery in MPS patients are associated with airway obstruction, with accompanying difficulty in ventilation and oxygenation, resulting in significant cardiovascular compromise.

Following is a list of serious anaesthetic complications that may occur during anaesthesia in patients with MPS:

- Inability to ventilate or intubate

- Temporary airway obstruction: can cause negative pressure (potentially obstructive) pulmonary oedema

- Complete airway obstruction (mostly during induction or at extubation): can cause profound hypoxaemia and cardiac arrest

- Post-intubation problems:

- Stridor

- Lower airway collapse/infection

- Need for reintubation or tracheostomy

The difficulty to ventilate using a face mask or difficulty or inability to intubate [can’t intubate, can’t ventilate (CICV)] is well illustrated by a case report discussing anaesthesia care in a 10-year-old boy with MPS IH (online supplementary material 1) (Kurdi and Deshpande 2008). A study including 34 patients who underwent 89 anaesthetics for 110 operations showed intubation difficulties in 11/29 patients who were intubated and failed intubation in three of them (Walker et al. 1994). Difficult intubations were documented in all MPS types included in the study [MPS IH, IHS (MIM ID #607015), IS (MIM ID #607016), II, IV (MIM ID #253000/10) and VI (MIM ID #253200)], but particularly common in MPS IH. A recent study including 17 MPS patients reported difficult mask ventilation in 20/141 anaesthetics (14.2 %), difficult intubation in 25 % and failed intubation in 1.6 % (Frawley et al. 2012). Older patients had more difficult intubations, confirming findings from previous studies (Belani et al. 1993; Moores et al. 1996). Sometimes, failure to intubate requires an emergency tracheostomy (Moores et al. 1996).

Extubation represents another major anaesthetic risk factor in MPS patients, who can develop postobstruction pulmonary oedema or be unable to maintain an airway after late extubation, requiring urgent reintubation or tracheostomy (Frawley et al. 2012; Walker et al. 2003b).

## Identifying anaesthetic risk factors in MPS

**Fig. 1:**
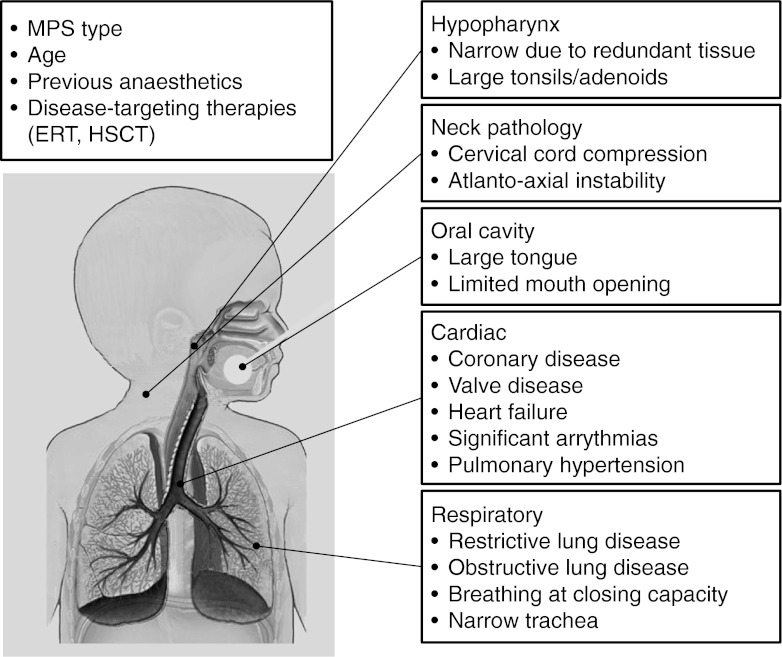
Overview of anaesthetic risk factors in patients with mucopolysaccharidosis (MPS).* ERT* enzyme replacement therapy,* HSCT* hematopoietic stem-cell transplantation

When planning surgery in MPS patients, it is important to weigh the benefits against the risks of the procedure (Walker et al. 1994). Anaesthetic risk factors should be carefully evaluated preoperatively (Fig. 1).

### Airway obstruction

The upper airway in MPS patients can be narrowed due to accumulation of GAGs, causing macroglossia, adenotonsillary hypertrophy and thickened soft tissues in the laryngopharynx. Progressive upper airway obstruction can be compounded by deformities of the skull or spine, such as a flattened nasal bridge, short neck, high anterior larynx, mandibular abnormalities or abnormal cervical vertebrae (Alpöz et al. 2006; Leighton et al. 2001; Myer 1991; Simmons et al. 2005). Multilevel airway obstruction may occur if upper airway obstruction is accompanied by tracheobronchomalacia or accumulation of GAG in the tracheal mucosa (Ingelmo et al. 2011; Leighton et al. 2001; Nagano et al. 2007; Pelley et al. 2007; Shih et al. 2002; Sims and Kempiners 2007). GAG accumulation in the larynx can impede identification of the glottis. Airway obstruction can also occur due to tracheal distortion in combination with laxity of tracheal tissue (Pelley et al. 2007; Walker et al. 2003a). Excessively thick secretions throughout the upper and lower airways can worsen airway obstruction. The degree of snoring or airway obstruction while sleeping is a very important part of the clinical history. MPS patients who have a history of severe obstructive sleep apnoea (OSA) are at high risk of airway emergencies during anaesthesia. OSA occurs in >80 % of patients (John et al. 2011; Leighton et al. 2001; Semenza and Pyeritz 1988).

Preoperative obstructive symptoms are a good indicator of postextubation respiratory difficulty (Belani et al. 1993). They may also predict intubation difficulties. The airway can be examined preoperatively for signs of obstruction using flexible nasoendoscopy under local anaesthesia in an awake patient. However, this requires a degree of cooperation and is not appropriate for all MPS patients (Kamin 2008). Alternatively, computed tomography (CT) of the thorax can be used, but this may require sedation or general anaesthesia. In a study by Ingelmo et al., anaesthetists changed their primary airway device selection after reviewing multidetector CT 3D images of the airways of children with MPS in 21 % (26/126) of evaluations (Ingelmo et al. 2011). Multidetector CT could provide additional information about glottic and subglottic structures, reduction and irregularities in the tracheal lumen and tracheomalacia. Dynamic CT studies can exclude the presence of dynamic tracheomalacia and evaluate the extent of a stenosis (Ingelmo et al. 2011; Murgu and Colt 2006). Mask continuous positive airway pressure may be required to keep the trachea open following extubation after anaesthesia.

### Respiratory abnormalities

MPS patients often develop restrictive pulmonary disease due to thoracic-cage abnormalities or compromised excursion of the diaphragm due to an enlarged liver and/or spleen or compromised neuromuscular function (Buhain et al. 1975; Giugliani et al. 2007; Leighton et al. 2001). Obstructive lung disease and diffusion defects have also been suggested to contribute to respiratory compromise (Semenza and Pyeritz 1988). Restrictive pulmonary disease, often in combination with airway obstruction, can lead to OSA, hypoventilation, pulmonary hypertension, cor pulmonale and eventually respiratory failure (Walker et al. 2003a).

Respiratory dysfunction can be highly disproportional to the patient’s clinical appearance. It can be detected preoperatively using respiratory function testing (e.g. spirometry), but these tests are difficult to interpret in MPS patients due to lack of reference data. Standard reference equations based on data from normal individuals may not apply to MPS patients with systemic skeletal dysplasia and extremely short stature. Cooperation problems due to young age, cognitive impairment or behavioural problems may also complicate respiratory function testing. The quality of results obtained by spirometry largely depends on the equipment used and the experience of the operator, particularly in children. A review by a pulmonologist may be very useful preoperatively to optimise respiratory function. The treatment of underlying chronic infection or bronchospasm is important, and preoperative investigation into the severity of OSA can be useful.

### Spinal cord risk

Spinal cord compression may occur due to spinal canal narrowing at the cervicocranial and thoracolumbar regions in patients with MPS I, II, IV, and VI (Tandon et al. 1996; Thorne et al. 2001). Patients with MPS IV, and to an extent those with MPS VI, are at risk of atlantoaxial instability due to odontoid hypoplasia (Ransford et al. 1996; Sims and Kempiners 2007; Walker et al. 1994, 2003b; McLaughlin et al. 2010). In patients with unstable necks, neck movement during intubation can lead to subluxation at the atlantoaxial region causing spinal cord damage and paralysis (Valayannopoulos et al. 2010; Walker et al. 1994). Spinal cord compression can also occur during long procedures or procedures requiring head movement (such as oral surgery). Therefore, a careful neurological examination (assessment of hyperreflexia), MRI scan of the spine in a neutral position and a flexion/extension X-ray of the spine can be recommended before the procedure to assess the risk of spinal cord compression.

### Cardiac risk

Cardiac manifestations are common in all types of MPS (Braunlin et al. 2011; Dangel 1998; Fesslová et al. 2009; Leal et al. 2010; Wippermann et al. 1995). Therefore, it is important to assess cardiac risk prior to general anaesthesia. Results of the cardiac consultation should be communicated to both surgeon and anesthesiologist. The cardiac consultation should include assessment of current haemodynamic stability, provide recommendations on the need for additional medications or tests, suggest the level of postoperative care and may even uncover a need to defer the procedure (Fleisher et al. 2009). Preoperative cardiac assessment should identify any cardiac disease that would place the patient in the surgical high-risk category. Cardiac conditions that affect operative mortality in the general population include unstable coronary syndromes, decompensated heart failure, significant arrhythmias and severe valvular disease (Fleisher et al. 2009). All these conditions can occur in MPS patients and warrant cardiac evaluation (Braunlin et al. 2011).

Identifying MPS patients who may be at increased risk of having myocardial ischemia during anaesthesia can be very difficult, as clinical signs may be masked preoperatively due to patient inactivity and communication difficulties. Coronary angiography can detect completely obstructed coronary vessels (van den Broek et al. 2011) but may underestimate severe diffuse coronary artery disease, which can result in unexpected sudden cardiac death during anaesthesia (Braunlin et al. 1992; Belani et al. 1993). Echo evaluation of left ventricular function during stress induced by intravenously administered dobutamine, which unmasks underperfused areas of myocardium, is noninvasive and safe in the general population but not yet routine in MPS (Kimball 2002). Severe coronary artery disease can be assumed to be present, especially if there is evidence for ischaemia on the 12-lead echocardiogram (ECG).

Decompensated heart failure associated with systolic dysfunction has been reported for different types of MPS (Fesslová et al. 2009; Hirth et al. 2007; Martins et al. 2009; Miller and Partridge 1983; Mohan et al. 2002; Wraith et al. 2008). MPS patients can also have diastolic dysfunction from hypertrophied myocardium and may respond poorly to fluid challenge. For patients with suspected cardiomyopathy, the ejection fraction and B-type natriuretic peptide are important as baseline measures and as a means of assessing response to therapy.

The spontaneous development of complete atrioventricular block has been described for MPS types II, III and VI (Dilber et al. 2002; Hishitani et al. 2000; Misumi et al. 2010). Symptoms of syncope or presyncope and evidence of any potential rhythm disturbance on ECG should be evaluated. The presence of PR interval at the upper limits of normal for age merits further investigation, such as Holter monitoring.

**Fig. 2:**
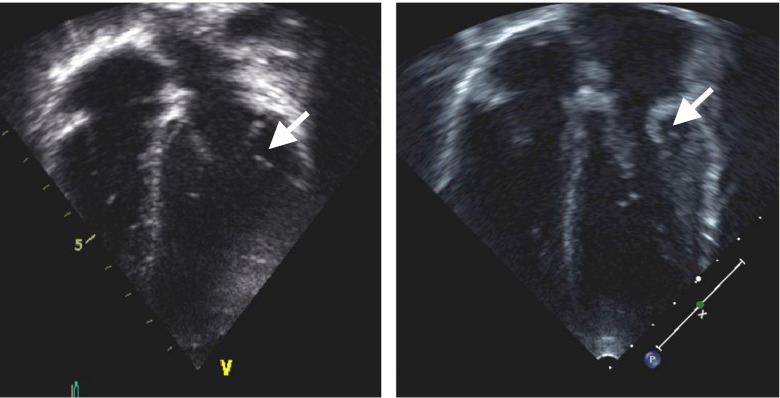
Echocardiographic images showing a normal mitral valve (*left*) and a thickened mitral valve in a patient with mucopolysaccharidosis (MPS) VI (*right*)

Cardiac valve disease is the most common cardiac finding in MPS. Progressive valve thickening from GAG deposition causes valve regurgitation and/or stenosis (Fig. 2), with mitral and aortic regurgitation being most common (Wippermann et al. 1995). Colour flow and Doppler interrogation of cardiac valves during cardiac ultrasound determines the severity of valve disease based on published guidelines (Bonow et al. 2008) and should be performed routinely in MPS. Severe aortic or mitral stenosis increases mortality risk during operative procedures in non-MPS patients and can also be expected to increase risk in MPS patients (Fleisher et al. 2009).

MPS patients, especially those who are untreated and have OSA, may develop pulmonary hypertension due to chronic hypoxaemia (John et al. 2011; Leal et al. 2010). The use of dopamine and other cardiovascular infusions should be considered when managing a patient with significant depression of cardiac ejection fraction. The use of intra-arterial cannulae for close monitoring of blood pressure should be considered for cases in which surgery may take a long time or for high-risk surgeries.

### Other risk factors specific for MPS

The type and severity of MPS are important indicators for anaesthetic risk. The most severe anaesthetic problems can be expected in patients with MPS IH, IHS, II, IV and VI. Because of the progressive nature of MPS, age is also an important anaesthetic risk factor (Orchard et al. 2010). Other factors to consider preoperatively are previous anaesthetics and treatment with hematopoietic stem-cell transplantation (HSCT) or enzyme replacement therapy (ERT) (Walker et al. 1994). Mouth opening or temporomandibular-joint range of motion should be measured, as some patients can only open their mouth a few centimetres (which may complicate intubation). Additionally, the tissues may be very thickened, stiff and immobile, especially in patients with MPS I and II.

## Anaesthetic management of MPS patients

### Preoperative preparation

Evaluation of anaesthetic risk factors before surgery allows the anaesthetist to anticipate problems that may arise during or after surgery (Online supplementary material 2). The potential risks of an operation should also be discussed with patients and their families, who should be involved in the decision whether or not to initiate the procedure (Walker et al. 1994). Preoperative sedation or premedication can be very useful to allay anxiety, especially as many patients are extremely anxious. The orally administered sedatives midazolam and diazepam are effective and will often have a good effect in a slightly reduced dosage. Orally available drying agents, such as atropine or glycopyronium, can be particularly useful to reduce secretions and improve the view when performing fiberoptic bronchoscopy or intubation. Patients should be monitored with a pulse oximeter (with a nurse present to monitor) once they have received a premedication. The occurrence of somnolence may indicate hypoxaemia if the patient has OSA; in this case, early intervention by the anaesthetist can prevent any further harm.

### Induction and intraoperative management

Normally, surgery in MPS patients requires general anaesthesia, although local anaesthesia may be an option for older patients with normal intelligence (Walker et al. 1994). General anaesthesia is preferably performed by a (paediatric) anaesthetist experienced in working with MPS patients (Kamin 2008). The anaesthetist should be surrounded by an experienced team and have access to all equipment and support that may be required (e.g. ENT specialist in case of a difficult airway, and intensive care backup). In addition to evaluation of airway obstruction, OSA, cardiac disease and pulmonary function, preoperative investigations should include an estimation of haemoglobin, serum electrolytes, oxygen saturation and, if indicated, X-rays of the chest and cervical spine (Walker et al. 1994). It is advisable to discuss the anaesthesia plan with the team before the start of the procedure, which often results in a Plan A and a backup Plan B. For instance, if mask induction followed by tracheal intubation orally proves unsuccessful, a fiberoptic intubation could be a backup plan. In a worst-case scenario, an emergency tracheostomy may be necessary. A backup plan allows preparation of the team and all (potentially) necessary equipment and a smoother procedural course.

Any sedation, whether it is inhalational or intravenous, might cause severe hypoxaemia due to airway obstruction in MPS patients (Online supplementary material 3). Using nitrous oxide to sedate a patient in order to facilitate placement of an intravenous catheter can be a safe alternative. Once the catheter is in situ, a careful induction with sedative agents can occur. Midazolam and fentanyl have been used successfully and can both be reversed with flumazenil and naloxone, if required. Both agents might cause respiratory depression and must be used with extreme caution. Ketamine can maintain a better airway at light levels of anaesthesia, allowing fiberoptic intubation without significant airway obstruction. Spontaneous ventilation techniques using oxygen and a high-concentration volatile anaesthetic have also been commonly used but require a skilled team to clear the airway if airway obstruction increases. Insertion of a laryngeal mask airway (LMA) will often improve ventilation, as will a nasal airway.

MPS patients can develop ventilation and intubation difficulties during induction (or after administration of muscle relaxants), requiring emergency tracheostomy (Moores et al. 1996). Muscle relaxants are best omitted until endotracheal intubation has been achieved and the airway is secure. Placing the patient in a lateral position can also help maintain the airway during the induction phase. This is often the preferred sleeping position for MPS patients with OSA, as in the supine position, but not the lateral position, gravity allows the tongue to obstruct the airway. In case of a difficult airway, intubation of the trachea should preferably be done using a fiberoptic bronchoscope. A good airway and sufficient time to do a bronchoscopy may be obtained using an LMA (Walker et al. 1994, 1997) (Online supplementary material 3). After the bronchoscope has passed into the trachea, a J-tipped guidewire can be passed down the suction channel of the bronchoscope into the trachea, the bronchoscope can be removed and a ureteral dilator or airway exchange catheter railroaded over the wire. Subsequently, the LMA can be removed and endotracheal intubation completed (Walker et al. 1997). An alternative approach for fiberoptic intubation is to intubate via the nose. Good preparation of the nose using a vasoconstrictor is essential to avoid bleeding into the airway (which could make the intubation more difficult).

Tracheal intubation problems frequently occur in MPS patients due to thickened and stiff tissues in the laryngopharynx and trachea, which can hamper access to the larynx using conventional equipment. The most appropriate uncuffed endotracheal tube is often two or three sizes smaller than predicted for age. An alternative approach is to use a small-sized paediatric cuffed endotracheal tube. It is essential to use the appropriate tube size to intubate in order to prevent extubation problems (Walker et al. 2003b).

Extubation can be problematic in patients with advanced clinical manifestations, especially in MPS I, II and VI. Preparation for extubation should include use of intraoperative steroids, full reversal of the muscle relaxant and placement of a nasopharyngeal airway to reduce upper airway obstruction after extubation. Extubation should be performed in an area where the patient can be reintubated as necessary and where all essential fiberoptic equipment and specialised personnel are available. In the high-risk patient, ENT support should also be available in case the patient fails extubation and requires a tracheostomy. A carefully positioned tube changer introduced via the endotracheal tube can be used to allow reintubation. This serves as a bridge device when successful extubation is uncertain; however, its use may promote obstruction and airway irritation in an already severely narrowed airway.

Anaesthesia for short procedures can be performed either using a face mask with a spontaneous breathing technique or an LMA. Intubation is not always necessary for short procedures, thus avoiding intubation and extubation difficulties. The use of a facemask can be difficult due to anatomical issues or excessive secretions (Frawley et al. 2012; Walker et al. 1994) or mucosal swelling after intubation.

### Postoperative care

Postoperatively, continued airway management and monitoring to detect airway obstruction episodes and desaturation is recommended until the patient regains full consciousness. Extubation should not be performed before the patient is fully awake, coughing vigorously, breathing adequately and moving deliberately (Walker et al. 2003b). Patients are best extubated early after surgery. This allows early assessment of neurological status and reduces airway swelling from intubation. If postoperative intubation is required for several days, fibreoptic bronchoscopy can be used to assess the extent of any swelling of the laryngeal area or obstruction from blood clots or other debris. As before, an adequate respiratory effort, a leak around the endotracheal tube and other measures necessary to ensure safe extubation should be followed.

In transoral approach to the anterior cervical spine, significant postoperative buccal swelling may occur. Therefore, a tracheostomy may be performed in advance. When posterior cervical spine surgery or thoracolumbar deformity correction surgery is performed, the child may remain prone for a prolonged period of time. Oral mucosal and tongue swelling may ensue, causing difficulty with breathing by a heavily swollen tongue, particularly in MPS IV and VI patients. The use of a steroid buccal cream helps reduce postoperative swelling. Postoperative ventilation or even tracheostomy may be necessary to allow tongue swelling to subside.

### Special considerations

Patients with potentially unstable necks require induction of anaesthesia with minimal or no neck movement using manual in-line stabilisation in order to prevent spinal cord damage (Valayannopoulos et al. 2010; Walker et al. 1994). This may complicate conventional direct laryngoscopy. Therefore, an alternative intubation technique is preferred, either using fiberoptic intubation or, potentially, a video laryngoscope (Theroux et al. 2012).

Spinal surgery at any level is associated with a higher risk of spinal cord injury. Therefore, neurophysiological monitoring using somatosensory or motor evoked potentials (SSEPs or MEPs) during surgery is required to monitor spinal cord function. This allows identification of surgery- or anaesthesia-induced neurophysiological changes, suggesting changes to the perfusion or direct damage to the spinal cord. Early recognition may prevent permanent damage and neurological deficit. Assessment of MEPs is essential in patients requiring instrumentation at the cervical or lower spine.

Neurophysiological monitoring may also be considered in patients undergoing procedures other than spinal surgery, particularly patients at increased risk of spinal cord compression and for long procedures or procedures requiring head movement (Linstedt et al. 1994; Sims and Kempiners 2007). In these cases, precautions should also be taken to maintain a neutral position during intubation and the procedure. Because of the increased risk of perioperative morbidity and mortality, these patients should be managed by experienced anesthesiologists at centres familiar with MPS disorders. Spinal cord compression may also occur in the absence of clinical neurological symptoms and after several hours of surgery (Linstedt et al. 1994). When neurophysiological monitoring is planned, total intravenous anaesthesia should be used, in view of dose-related alterations in SSEP with volatile anaesthetics. Inhalational anaesthetics diminish the ability to obtain MEPs and cortical responses from SSEP. However the use of volatile aesthetics at less than one minimum alveolar concentration (MAC) supplemented with remifentanil could be considered during SSEP. Subcortical responses can be monitored in the presence of inhalational anaesthetics using SSEP.

## Management of emergency airway issues in MPS

The most important anaesthetic issues in MPS are related to airway obstruction and may occur irrespective of the length or complexity of the procedure. Airway obstruction during induction can occur early in patients with advanced clinical manifestations and can lead to significant hypoxaemia and cardiac arrest or to obstructive pulmonary oedema (Walker et al. 2003b). Treatment would normally consist of intubation and positive pressure ventilation. However, patients may be very unstable and develop profound oxygen desaturation, making intubation difficult or impossible, sometimes requiring emergency tracheostomy. Postobstructive pulmonary oedema can also develop after failed extubation (Walker et al. 2003b). This has been reported after cervical fusion surgery after the application of a halo thoracic jacket. In this situation, reintubation can be very challenging.

Performing a surgical tracheostomy in MPS patients can be difficult due to the short neck, thickened soft tissues and the resultant deep position of the trachea within the neck. An emergency surgical tracheostomy will therefore take longer than usual, and every effort should be made to avoid this scenario with its significant potential to threaten life. A percutaneous tracheostomy is highly unlikely to be successful due to the factors previously mentioned (Pelley et al. 2007). A tracheotomy may also be performed in a more elective situation; for example, in a patient requiring major surgery who needs postoperative intensive care (Online supplementary material 2). Some institutions believe that leaving an endotracheal tube in place for a prolonged period may lead to additional postintubation changes in the airway, which will exaggerate existing airway obstruction, significantly limiting the potential success of attempted extubation (Jeong et al. 2006; Muhlebach et al. 2011; Pelley et al. 2007; Sims and Kempiners 2007). An emergency cricothyrotomy is to be discouraged, as identifying and accessing the trachea, passing a needle through the cricoid cartilage and advancing an airway device is a challenge for the most skilled surgeons.

## Future of MPS management and implications for the anaesthetist

The life expectancy of MPS patients has markedly increased with the introduction of ERT and HSCT. These therapies have a positive impact on pulmonary function, which may also reduce the anaesthetic risk. HSCT in MPS I patients before the age of 2 years appears to reduce airway complications and improve mask ventilation and intubation (Frawley et al. 2012; Kirkpatrick et al. 2012). However, the use of HSTC is limited in MPS types other than MPS IH (Rovelli 2008). ERT (generally initiated late in the clinical course) improved upper airway patency in MPS II and MPS VI patients (Frawley et al. 2012). The increased life expectancy associated with ERT and HSCT goes along with an increased demand for surgery and anaesthesia. A rising number of older patients require repeat procedures for, e.g., intravenous access devices and neuro-, orthopaedic or palliative surgery. Ageing can be associated with severe narrowing of the larynx or trachea and often with severe OSA, which poses significant challenges to the anaesthetist.

Due to the rarity of MPS, most anaesthetists are not familiar with the anaesthetic issues associated with the disease. Therefore, anaesthetic and surgical care of these rare disorders is preferably concentrated in one or two centres per region or country. Training using flexible bronchoscopy simulators can be valuable to allow residents and anaesthetists to learn more about airway anatomy and to practice psychomotor skills and fiberoptic intubation procedures in a safe environment. In this way, they can be prepared to act adequately in difficult (e.g. MPS) patients and exceptional situations (Rowe and Cohen 2002). In the future, it may become possible to load a CT scan or MRI of a patient to the simulator and practice intubation before the operation. This will allow the anaesthetist to anticipate potential problems that may occur during anaesthesia.

## Conclusions

The MPS patient poses a major challenge to the anaesthetist. The anaesthetic risk can be reduced considerably if the anaesthetist anticipates potential problems that may arise in these patients during and after the procedure, including difficult intubation and ventilation, and cardiac and cervical spine issues. This requires a thorough preoperative evaluation and knowledge of the pathophysiology underlying the respiratory and cardiac manifestations, as well as cervical and tracheolaryngeal anatomy in these patients. Therefore, these difficult decisions should ideally be made by a multidisciplinary team in a tertiary referral centre experienced in the perioperative management of MPS patients. Anaesthesia in patients with an unstable spine or for spine surgery is particularly difficult and requires additional care and thought in the choice of anaesthetic, monitoring and postoperative care.

## Electronic supplementary material

Below is the link to the electronic supplementary material.

Fig. 1 Case report from a boy with MPS) IH illustrating intubation difficulties in such patients (Kurdi and Deshpande 2008).* ENT* ear-nose-throat,* LMA* laryngeal mask airway (PDF 16 kb)

Fig. 2 Case report illustrating preoperative evaluation and anaesthesia in a 15-year old boy with mucopolysaccharidosis (MPS) VI.* CT* computed tomography,* OSA* obstructive sleep apnoea (PDF 23 kb)

Fig. 3 Case report illustrating anaesthetic problems due to airway obstruction and management in a 19-year old girl with mucopolysaccharidosis (MPS) IHS.* LMA* laryngeal mask airway (PDF 32 kb)
