# Supplementary material for: Anaesthesia and airway management in mucopolysaccharidosis
Source: J Inherit Metab Dis. 2012 Nov 30;36(2):211–9. doi: 10.1007/s10545-012-9563-1 (PMC3590422; doi:10.1007/s10545-012-9563-1)
Supplement: Supplementary file 1 — Case report from a boy with MPS) IH illustrating intubation difficulties in such patients (Kurdi and Deshpande 2008). ENT ear-nose-throat, LMA laryngeal mask airway (PDF 16 kb) [file 10545_2012_9563_MOESM1_ESM.pdf]

MPS IH

- 10-year-old boy scheduled for emergency umbilical hernia repair
- First attempt of anaesthesia failed:
- failure of ventilation with a facemask after a muscle relaxant was given
- intubation failure with a 5 mm conventional endotracheal tube
- Examination of ENT manifestations by indirect laryngoscopy not possible because patient did not cooperate following procedure
- Second attempt 2 days later:
- Consent for tracheostomy was obtained and LMA and a senior anaesthetist were on standby
- Several unsuccessful attempts for oral intubation with a 5 mm tube and smaller tube sizes □ blind nasal intubation with an endotracheal tube (successful)
- Continuation of surgery
