# Supplementary material for: Anaesthesia and airway management in mucopolysaccharidosis
Source: J Inherit Metab Dis. 2012 Nov 30;36(2):211–9. doi: 10.1007/s10545-012-9563-1 (PMC3590422; doi:10.1007/s10545-012-9563-1)
Supplement: Supplementary file 2 — Case report illustrating preoperative evaluation and anaesthesia in a 15-year old boy with mucopolysaccharidosis (MPS) VI. CT computed tomography, OSA obstructive sleep apnoea (PDF 23 kb) [file 10545_2012_9563_MOESM2_ESM.pdf]

[redacted]

**Clinical background and preoperative evaluation**

- 15-year old boy, 35 kg, with long tract signs from compression of the cervical spine
- History of noisy breathing and OSA
- Pre-operative CT thorax showed narrowing at laryngeal level and lower in the thorax
- Procedure initially delayed due to concern of tracheal stenosis
- Preoperative plan: examine fiberoptically and decide whether to proceed

**CT thorax and bronchoscopy**

- Glottic narrowing
- Kinking in trachea

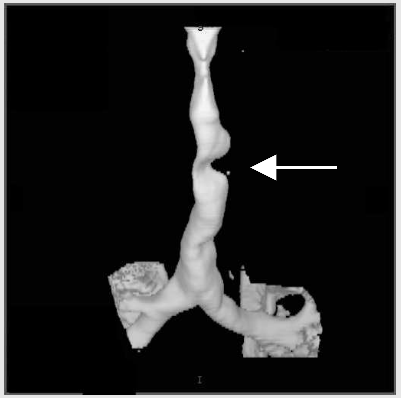

**Between evaluation and procedure**

- 3 episodes of collapse, 2 requiring resuscitation, over 3 months

**Procedure**

- Anaesthetised in lateral position with sevoflurane in oxygen
- Fiberoptic intubation using a small plain tube
- Tracheostomy performed with the tip placed distal to the tracheal narrowing
- Procedure carried out and good recovery
