# Supplementary material for: Anaesthesia and airway management in mucopolysaccharidosis
Source: J Inherit Metab Dis. 2012 Nov 30;36(2):211–9. doi: 10.1007/s10545-012-9563-1 (PMC3590422; doi:10.1007/s10545-012-9563-1)
Supplement: Supplementary file 3 — Case report illustrating anaesthetic problems due to airway obstruction and management in a 19-year old girl with mucopolysaccharidosis (MPS) IHS. LMA laryngeal mask airway (PDF 32 kb) [file 10545_2012_9563_MOESM3_ESM.pdf]

MPS IHS

Clinical background

- 19-year old girl, small stature, 25 kg, presented for corneal graft surgery
- Pre-operative fiberoptic bronchoscopy on 3 occasions in previous 3 years showed thickened and narrowed glottic anatomy
- Recent decompression of spinal cord at craniocervical junction: patient remained intubated and ventilated for 3 days, followed by successful extubation
- Increasing hoarsness on presentation for corneal graft surgery

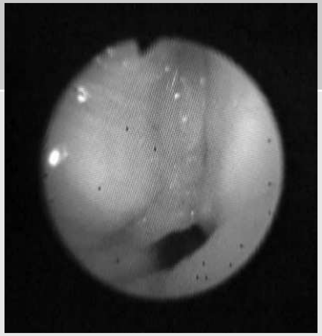

Endoscopic image showing thickening of larynx (laryngeal opening 3-4 mm)

Procedure

- General anaesthesia using fiberoptic intubation and inhalation induction
   Airway deteriorated after loss of conciousness
   insertion of LMA size 2 first and then changed to size 2.5 resulting in good O2 saturation, but persistent degree of airway obstruction
   fiberoptic bronchoscopy: revealed extremely thickened larynx
   procedure abandoned to prevent further swelling

- Second trial of anaesthesia: same procedure, but airway less obstructed
   LMA size 2.5 placed when level of anaesthesia was sufficient and airway seemed relatively unobstructed (required some chin lift with LMA in place)
   again, bronchoscopy showed extremely narrowed airway
   glottis sprayed via suction channel of fiberoptic scope with lidocaine, followed by 3 mL of 1/10,000 epinephrine to shrink structures
   after a few minutes, trachea could be entered succesfully, a guide wire was placed, and a Cook airway exchanger railroaded over the guide wire after removal of the bronchoscope
   size 4.5 plain endotracheal tube railroaded over exchange catheter, but replaced by 4.5 mm microcuff endotracheal tube due to substantial leak
   after 2 mL of air cuff, successful ventilation of patient albeit with a small leak
   dexamethasone given during surgery to reduce any further glottic swelling
   successful extubation in anaesthetic room and discharged home 1 day later
